# Intelligent systems in obstetrics and midwifery: Applications of machine learning

**DOI:** 10.18332/ejm/143166

**Published:** 2021-12-20

**Authors:** Stavroula Barbounaki, Victoria G. Vivilaki

**Affiliations:** 1 Department of Midwifery, School of Health and Care Sciences, University of West Attica, Athens, Greece

**Keywords:** diagnosis, pregnancy, obstetrics, midwifery, machine learning, intelligent systems

## Abstract

**INTRODUCTION:**

Machine learning is increasingly utilized over recent years in order to develop models that represent and solve problems in a variety of domains, including those of obstetrics and midwifery. The aim of this systematic review was to analyze research studies on machine learning and intelligent systems applications in midwifery and obstetrics.

**METHODS:**

A thorough literature review was performed in four electronic databases (PubMed, APA PsycINFO, SCOPUS, ScienceDirect). Only articles that discussed machine learning and intelligent systems applications in midwifery and obstetrics, were considered in this review. Selected articles were critically evaluated as for their relevance and a contextual synthesis was conducted.

**RESULTS:**

Thirty-two articles were included in this systematic review as they met the inclusion and methodological criteria specified in this study. The results suggest that machine learning and intelligent systems have produced successful models and systems in a broad list of midwifery and obstetrics topics, such as diagnosis, pregnancy risk assessment, fetal monitoring, bladder tumor, etc.

**CONCLUSIONS:**

This systematic review suggests that machine learning represents a very promising area of artificial intelligence for the development of practical and highly effective applications that can support human experts, as well the investigation of a wide range of exciting opportunities for further research.

## INTRODUCTION

In the past few years, interest has grown regarding the use of artificial intelligence (AI) techniques in the field of medicine in general and in midwifery and obstetrics in particular^1^. In parallel with the technological progress experienced worldwide, AI capabilities have largely improved, as new learning algorithms, theories and advanced computing power came into play, bridging the gap from theoretical models to practical use^2^. Machine learning (ML), a subcategory of AI which basically teaches computers to perform tasks on their own without explicit implementation of rules, has experienced changes of similar magnitude. Machine learning (ML) encompasses methods of data analysis in order to produce models that can be used to represent and solve problems in several domains. In the context of ML methods, algorithms are developed and utilized to produce knowledge straight from data analysis. ML produces knowledge as more data are fed into the ML algorithms. ML, therefore, makes it possible for more concepts, i.e. variables to be included into a model and more associations to be specified. Such variables or associations are not necessarily known or eligible for model inclusion before data analysis with ML. Artificial Intelligence (AI) approaches, in general, have attracted a lot of attention over recent years because of their ability to tackle problems where conventional approaches have either failed or have not been so effective. Central to AI, and of course to ML importance increase, is the vast amount of data that are available today, which is attributed mainly to the expansion of the internet and e-services as well as to the imminent development of the Internet of Things (IoT). ML assumes two main approaches to learning, namely supervised and unsupervised learning. Supervised learning considers a labelled set of data with clearly marked input and output values. The purpose of ML is training the appropriate algorithm with a data set to devise a plan for producing the anticipated output given a set of input data. In the case of the unsupervised learning a suitable algorithm is trained on unlabeled and unclassified data, allowing the computer to group data in light of likeness or contrast.

## METHODS

A thorough literature analysis in four electronic databases (PubMed, APA PsycINFO, SCOPUS, ScienceDirect) was performed that produced a set of articles selected for further consideration. This systematic review was done according to the Systematic Reviews and Meta-Analysis (PRISMA) method. As for the studies eligibility criteria, the literature review considered only primary studies in English without geographical limitations, published during the period 2012–2021 and relevant to machine learning applications and intelligent systems in midwifery and obstetrics domains.

Searching strings were: [‘machine learning’ OR ‘intelligent systems’] AND [‘obstetrics’ OR ‘midwifery’ OR ‘pregnancy’ OR ‘pregnancy risks’ OR ‘perinatal distress’ OR ‘postpartum period’ OR ‘fetal’ OR ‘breast feeding’ OR ‘cervical’].

This review focuses on studies that only discuss machine learning methods and intelligent systems applied in the obstetrics and midwifery domains, in a broad range of topics. Selected research studies were all critically evaluated, and a contextual synthesis of results was performed.

**Figure 1:**
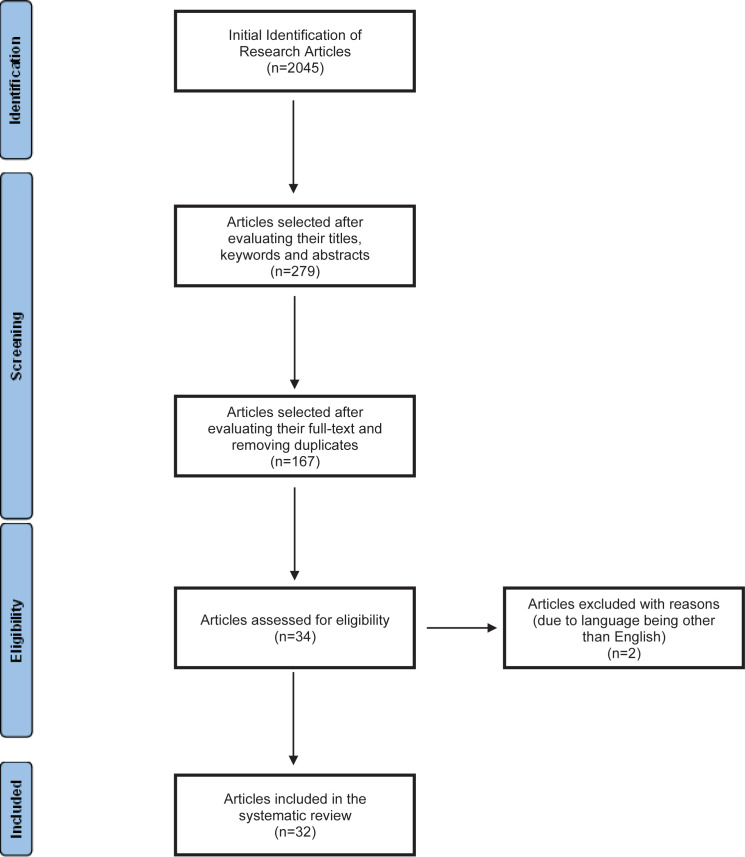
The process for identifying and selecting the articles for the systematic review

Search criteria initially returned 2045 research studies. Following the evaluation of the articles’ titles, keywords, and abstracts with respect to their relevance to this systematic review, 279 articles remained for further analysis. By screening the articles’ full-text and removing duplicates, 32 studies remained for this systematic review. Any disagreements that may have risen were resolved by discussion between the reviewers. The process followed for the identification, screening, and inclusion of the literature, is shown in Figure 1.

## RESULTS

Nowadays, ML is widely used in extensive datasets, as it is capable of tracing patterns that eventually lead to accurate predictions^2^. An example of the extensive use of ML lies in the domain of reproductive medicine (Assisted Reproductive Technology – ART). More specifically, ML engages in the process of decision making, predicting pregnancy outcomes, while also dealing in the most effective way with infertility. Surpassing traditional statistics, ML is not only used to rate disease conditions but most importantly to provide a medium for accurate predictions and treatments, something that can be achieved with the help of large amounts of training data^2^. Having this in mind, many researchers have recently tried to develop ML models in the field of medicine. The 32 studies included in this systematic review are presented in Table 1.

**Table 1:** The characteristics of the studies included in this systematic review

	*Field of interest*	*Problem domain in midwifery or obstetrics*	*Authors Year*	*Classifiers*	*Number of samples*	*Results*
**1**	**Implantation outcome of in vitro fertilization (IVF)**	Extract and discover patterns that provide knowledge regarding the implantation outcome of In vitro fertilization (IVF) and intracytoplasmic sperm injection (ICSI)	Hafiz et al.^3^ 2017	Random forest algorithm and recursive partitioning (RPART)	486 patients	Superior accuracy contrasted with other classification. Forecasting tools: 84.23% with Random Forest algorithm and 82.05% with RPART
**2**		Trace the best classifier to predict the implantation outcome of IVF	Uyar et al.^4^ 2015	Comparison of six classifiers – the Naïve Bayes classifier proved to be the best	2453 embryos	Accuracy level of 80.4%, sensitivity rate 63.7%, false-positive rate 17.6%
**3**	**Classificationof sperm cells**	Analysis of semen - classification of sperm cells (as normal or abnormal)	Goodson et al.^5^ 2017	Support vector machines (SVM) with multiclass Decision Tree (DT) to address the issue of sperm motility clustering	2817 sperm from 18 individuals	Accuracy level of 89.9%
**4**		Sperm morphology clustering	Tseng et al.^6^ 2013	SVM based model	160 human sperms	Precision level of 87.5%
**5**		Sperm morphology clustering	Mirsky et al.^7^ 2017	SVM	1405 sperm cells	Precision level of >90%
**6**		Automatic assessment of human blastocysts	Santos Filho et al.^8^ 2012	Combination of automated image analysis/ segmentation and SVMs	93 images of different blastocysts	Accuracy level of 67–92%
**7**		Forecast implantation	Milewski et al.^9^ 2017	Principal component analysis (PCA) and artificial neural network (ANN)	610 embryos’ morphokinetic information	Efficiency level of 75%
**8**		Identify the health of human sperm	Li et al.^10^ 2014	Combination of principal component analysis (PCA) and the k-nearest neighbor algorithm (KNN)	80 microscope images	Accuracy level of 95.73% regarding healthy human sperm and 51.35% regarding unhealthy sperm
**9**	**Embryo selection**	Segmentation of trophectoderm (TE) region and of the inner cell mass (ICM) of the blastocyst images	Saeedi et al.^11^ 2017	Segmentation algorithm	211 blastocyst images	Accuracy level of 86.6% concerning the recognition of TE and 91.3% concerning ICM
**10**		Predict the quality of embryos and oocytes and improve the performance of assisted reproduction technology	Manna et al.^13^ 2013	Neural Networks	Two data sets from 104 women. The one includes 269 photographs of oocytes and the other consists of 269 photographs	Authors claim the results clearly outperform the existing approaches
**11**		Automatic model for recognizing the trophectoderm (TE) region of human blastocysts	Singh et al.^14^ 2015	Retinex algorithm to distinguish the shapes of the images	85 images	Precision level of 87.8% concerning the identification of TE region
**12**	**Forecast vaginal delivery (VD) in twins**	Forecasting vaginal delivery (VD) in twins	Lumbreras- Marquez et al.^15^ 2021	RF algorithm employed with 12 predictors	1054 women	Sensitivity of 97%, specificity value of 20%. positive forecasting rate 80% and negative forecasting rate 67%
**13**	**Cervical cancer**	Forecasting cervical cancer patient’s survival outcome	Matsuo et al.^16^ 2019	Deep-learning neural network and Cox proportional hazard regression mode (40 predictors)	768 women	The Deep learning model revealed more accurate results concerning the forecasting of progression free-survival compared to the Cox proportional hazard regression model
**14**	**Rehospitalization of the mother due to hypertensive disorders of pregnancy**	Forecasting the re-hospitalization of the mother due to hypertensive disorders of pregnancy (predict a 42-day after delivery readmission)	Hoffman et al.^17^ 2021	Data from the electronic medical records	5823 pregnant women	Further investigation and exploitation of ML techniques could eventually prove beneficial
**15**	**Postpartum maternal hemorrhage (PPH)**	Estimating and predicting the risk of postpartum maternal hemorrhage (PPH)	Westcott et al.^18^ 2020	Regression- tree and Kernel ML techniques Data from the electronic medical records (471 variables)	30867 women	Gradient boosted decision trees models (XGBoost) performed best regarding postpartum hemorrhage classification (precision level of 98.1% with a sensitivity level of 0.763) when compared to Random Forest (precision level of 98.0% with a sensitivity level of 0.737)
**16**		Predicting postpartum hemorrhage	Venkatesh et al.^19^ 2020	Random Forest, Extreme Gradient Boosting models and statistical models (logistic regression with and without lasso regularization) (55 risk factors)	152279 births 7279 faced postpartum hemorrhage	Gradient Boosting model performed best (C statistic=0.93; 95% CI: 0.92–0.93), while the Random Forest model also achieved satisfactory results (C statistic=0.92; 95% CI: 0.91–0.92).
**17**	**Neonatal mortality prediction**	Predict neonatal mortality related to hypoxic- ischemic encephalopathy (HIE) – risk classification	Slattery et al.^20^ 2020	Neural networks (convolutional and two recurrent ones) using children’s hospital neonatal database	52 nonanomalous neonates	Specificity for Convolutional networks was 81% - for Recurrent models with long shortterm memory 69%, and for Gated recurrent model 65%
**18**		Identification of preterm newborns in low- and middleincome countries - neonatal mortality	Rittenhouse et al.^21^ 2019	Multiple parameter machine learning models	862 newborns	Results revealed a set of 6 maternal and newborn characteristics which could eventually lead to precise identification
**19**	**Fetal monitoring**	Predict the risk for euploidy, trisomy 21 (T21) and other chromosomal aneuploidies (O.C.A.)	Neocleous et al.^24^ 2016	Artificial Neural Networks (ANN)	Data set consists of 16898 cases of euploidy fetuses, 129 cases of T21 and 76 cases of (O.C.A.)	The ANN identified correctly all T21 cases and 96.1% of euploidies, meaning that no child would have been born with T21 if only that 3.9% of all pregnancies had been sent for invasive testing
**20**		Predict perinatal outcome in asymptomatic pregnant women with short CL	Bahado-Singh et al.^25^ 2019	Dep Learning	26 patients	Very good to excellent prediction rates (88.5% accuracy)
**21**		Trace abnormal fetal cardiac anatomy based on automatic echocardiography views	Yeo et al.^27^ 2013	A method (FINE) which revealed four correctly positive cases of abnormality	50 spatiotemporal image correlation (STIC) volume datasets	In all four abnormal cases, the FINE method demonstrated evidence of abnormal fetal cardiac anatomy
**22**		Distinction of hypoplastic left heart syndrome and normality (HLHS)	Arnaout et al.^28^ 2018	Convolutional DL method	685 echocardiograms	Specificity 100% and sensitivity 90%
**23**		Trace both obstetrical and fetal complications timely	Escobar et al.^29^ 2021	Automated electronic medical record (EMR) data – Gradient boosting-based model and logistic model	303.678 admissions and 239.526 eligible patients	Both models were rejected. Further analysis is proposed
**24**		Calculate fetal cardiac biometrics by identifying canonical screening views of fetal heart and segmenting cardiac structures	Arnaout et al.^28^ 2018	Convolutional DL method	685 echocardiograms	Sensitivity of 75% (100%) and specificity of 76% (90%) when distinguishing normal heart vs TOF (HLHS)
**25**		Obstetric and fetal complications using Automated Electronic Health Record Data	Escobar et al.^29^ 2016	Logistic regression and Gradient boosting	Data collected from 209611 randomly selected deliveries	Model produced Promising results but needs improvements
**26**	**Preeclampsia**	Prediction of preeclampsia occurrence	Jhee et al.^33^ 2019	Logistic regression, DT, Naive Bayes classification, SVM, RF algorithm and stochastic gradient boosting (SGB) methods	11006 expecting women	Stochastic gradient boosting (SGB) model proved to be adequate and showed the best performance (accuracy of 0.973 and false-positive rate of 0.009)
**27**		Forecast blastocyst formation using oocyte mechanical properties	Kort et al.^34^ 2018		773 oocytes	Positive predictive value of 80% and negative predictive value of 63.8%
**28**		Identification of good quality embryos	Iwata et al.^35^ 2018	Deep Learning prediction model with Keras neural network library framework	A wide range of sample sizes were used, e.g. 3 patients with 16 follicles, 118 embryos, 160 blastocysts, 223 embryo images	94% accuracy level for the training dataset and 70% for the validation dataset
**29**		Proper embryo selection	Tran et al.^36^ 2018	AI Deep neural network	A total of 10208 embryos from 1603 patients were extracted	Mean Area Under the Curve (AUC) of 0.93, 95% CI for predicting FH outcome
**30**	**Dystocia**	Forecast shoulder dystocia	Bartal et al.^37^ 2019	Using maternal demographic, obstetric history, and sonographic evaluation	490 patients	Fetal weight (EFW) assessment alone produced inferior outcomes compared to the combination of ML and EFW. Further research on the area could eventually prove beneficial
**31**	**Predicting successful vaginal deliveries**	Successful prediction of vaginal deliveries	Guedalia et al.^38^ 2020	Personalized ML-based prediction model and real-time data of the first stage of labor	94480 cases of vaginal deliveries	Further research and upgrading of personalized ML-based prediction models is necessary
**32**		Predict the chance of a successful vaginal delivery after the occurrence of a cesarian delivery	Lipschuetz et al.^39^ 2020	Two ML based submodels were created (one with data gathered from the first antenatal visit and another with added data available close to the delivery process)	9888 women with previous CD	The second model exhibited greater results than the first

## DISCUSSION

### Implantation outcome of in vitro fertilization (IVF)

Hafiz et al.^3^ employed machine learning techniques in order to extract and discover patterns that would eventually provide knowledge regarding the implantation outcome of IVF and intracytoplasmic sperm injection (ICSI). For this purpose, researchers gathered data from 486 patients and employed the random forest algorithm as well as recursive partitioning (RPART). When compared with other classification and forecasting tools, the accuracy of the specific technique in predicting the relevant outcomes, proved to be superior (84.23% and 82.05% for the two classifiers, respectively).

In similar research, Uyar et al.^4^ compared six classifiers, in order to trace the one that best predicts the implantation outcome of IVF (a process which according to them could be facilitated by implementing a constant on embryos’ morphological features). Eventually, the model with the best predictions proved to be Naïve Bayes. The Naïve Bayes classifier, which can also operate in small amounts of training data (as it calculates feature probabilities separately), provided decision support by taking into consideration the total number of embryos transferred. Percentages revealed an accuracy of 80.4%, a sensitivity rate of 63.7% and a false-positive rate of 17.6%, results that even surpassed the experts’ own opinions, without the help of ML techniques.

### Classification of sperm cells

Moving on to male factor infertility, various researchers have employed support vector machines (SVM), which have played a crucial role in the classification of sperm cells (as normal or abnormal) and the analysis of semen. Based on the element of sperm cells’ morphology, experts along with SVM are able to decide on the Assisted Reproductive Technology options (ART), whenever lower fertilizing potential occurs. Relevant research studies have been produced by many authors including Goodson et al.^5^ who developed a SVM along with a multiclass Decision Tree (DT) to address the issue of sperm motility clustering. The final accuracy of the model reached 89.9%. Giving attention to the same issue (sperm morphology clustering), Tseng et al.^6^ had previously proposed another SVM-based model, which used one-dimensional features extracted from 160 human sperms. The model revealed a precision level of 87.5%. Later on, Mirsky et al.^7^ attempted to train an SVM to perform the morphological clustering of 1405 sperm cells on its own, an experiment that also displayed good results (typical ROC curve=88.59%, area under precision-recall curve=88.67% and precision ≥90%). It should be noted though, that SVMs are also used in order to evaluate embryos’ viability. More importantly, when combined with automated image analysis, SVMs do not only assist in proper embryo assessment but also in the process of selection. Taking this into account, Santos Filho et al.^8^ put together image segmentation and SVMs to perform an almost automatic assessment of human blastocysts, a model that reached an accuracy level of 67–92%, suggesting that there is still plenty of work to be done in this field.

Unsupervised learning remains a terra incognita too. In this case, however, some investigators have already proposed certain theories and models. Milewski et al.^9^ for instance, employed principal component analysis (PCA) and artificial neural network (ANN) to forecast implantation by using 610 embryos’ morphokinetic information recordings moved in 514 cycles. The model achieved an efficiency level of 75% in the area under curve. Using similar techniques, Li et al.^10^ had proposed a combination of PCA and the k-nearest neighbor algorithm (KNN) in order to identify the health of human sperm. This model outperformed previously used ones with an accuracy level of 95.73% regarding healthy human sperm and 51.35% regarding unhealthy sperm.

### Embryo selection

Moving on to the issue of embryo selection, it is evident that it highly depends on the assessment of embryo viability^11^. Despite multiple efforts made, embryo viability remains a challenging task nowadays. As known, a successful implantation does not solely depend on the experts’ knowledge and experience, but also on existing embryo scoring systems^8,12,13^. Therefore, the need for a development of even more efficient AI systems is profound. Bearing this in mind, Singh et al.^14^, attempted to build an automatic model for recognizing the trophectoderm (TE) region of human blastocysts, using the Retinex algorithm, in order to distinguish the shapes of the images more clearly. When tested, this model achieved a precision level of 87.8% concerning the identification of TE regions. In the same direction, Saeedi et al.^11^ not only achieved segmentation of TE but also of the inner cell mass (ICM) of the blastocyst images; 211 blastocyst images were checked and the results indicated an accuracy level of 86.6% concerning the recognition of TE and 91.3% concerning ICM, rendering the combination of AI tools and embryo morphology a promising start in the fight against infertility.

### Forecast vaginal delivery (VD) in twins

Machine learning can also be incorporated in other similar matters. For instance, Lumbreras-Marquez et al.^15^ developed an ML-based model to forecast vaginal delivery (VD) in twins. For this purpose, researchers studied 1054 women. Meanwhile, the infused values among 17 predictors amounted to 14%, while the frequency of vaginal delivery reached 77%. The RF algorithm employed, chose 12 predictors to be included in the training data including gestational age, ART etc. Results revealed a 97% sensitivity, a 20% specificity, an 80% positive forecasting rate and a 67% negative forecasting rate, proving that ML-based models can also assist in patient guidance and labor monitoring, concerning twin pregnancies.

### Cervical cancer

Addressing an entirely different topic, Matsuo et al.^16^ compared different models in order to trace the one that works best in forecasting a cervical cancer patient’s survival outcome. A deep-learning neural network and a Cox proportional hazard regression model were developed in an effort to find the one that could predict the survival outcome with a higher degree of precision. The study concerned newly identified stage I-IV cervical cancer cases from 2000–2014; 40 predictors were evaluated (i.e. vital signs, treatment types etc.) and later on categorized into groups of three. The two aforementioned models were compared with 3 other survival analysis models as well; 768 women participated in the study (mean age 49 years, Hispanic in majority, squamous tumor type, and stage I in majority), while the monitoring time was estimated 40.2 months. During that period, 241 cases of recurrence were presented, as well as 170 deaths. It was found that the deep learning model revealed more accurate results concerning the forecasting of progression-free survival compared to the Cox proportional hazard regression model (mean absolute error 29.3 vs 316.2). It also presented higher levels of precision with regard to the prediction of overall survival (mean absolute error 30.7 vs 43.6). More interestingly, it was also observed that the deep learning model was further upgraded whenever feature addition occurred (i.e. concordance index as far as progression-free survival is concerned of 0.695 for 20 features and 0.795 for 40 features). It is evident therefore that the specific promising model can assist experts in the analysis and prediction of survival outcomes in the future.

### Re-hospitalization of the mother due to hypertensive disorders of pregnancy

ML techniques, however, have also been used by some researchers in order to provide solutions to other problems such as forecasting the re-hospitalization of the mother due to hypertensive disorders of pregnancy. This issue is of great importance as maternal mortality cases related to such disorders persist, while accurate prediction of readmission rates remains low. Hoffman et al.^17^ proposed an algorithm that could tackle this hazardous matter. Surveying a single institution, the investigators used data from the electronic medical records at the time of maternal discharge, to predict a 42-day after delivery readmission. Afterwards, they separated the data into a derivation (including 20.032 pregnant women with 238 readmissions/1.2%) and a validation cohort (including 5.823 pregnant women with 82 readmissions/1.4%). The model found 31 clinical features that could predict readmission in both cases, suggesting that further investigation and exploitation of ML techniques could eventually prove beneficial as it could predict the risk of re-hospitalization, resulting in early awareness of an imminent danger.

### Postpartum maternal hemorrhage

With an objective to predict the risk of postpartum maternal hemorrhage (PPH), Westcott et al.^18^ developed another model, based on regression-, tree- and kernel-ML techniques, in order to separate women into groups, based on the risk they have of getting hemorrhage; 30.867 women (from July 2013 to October 2018) participated in the study, while 471 variables were gathered from the electronic medical records, i.e. family history, vital signs etc. Two sub-models were created and compared; the first one included data from all stages of pregnancy, whereas the second one did not use much data prior to the stage of labor. Results indicated that the Gradient boosted decision trees models (XGBoost) performed best regarding postpartum hemorrhage classification, when compared to other models (i.e. Random Forest). Meanwhile, the first sub-model (including all data) achieved slightly greater accuracy (Area under Curve 0.979, accuracy 95%, Proper Classification of PPH 0.971–0.986) than the second one (including limited pre-labor data) (Area under Curve 0.955, accuracy 95%, Proper Classification of PPH 0.939–0.970). Overall, regarding positive predictions of PPH, the first model achieved a precision of 98.1% and a sensitivity of 0.763, whereas the second achieved values of 98.0% and 0.737, proving that an XGBoost based model could eventually work as an accurate prevention tool, assisting in timely diagnosis and patient counseling (including care transfer decisions).

Venkatesh et al.^19^ also created and compared ML based models (Random Forest and Extreme Gradient Boosting) and basic statistical ones (logistic regression with and without lasso regularization), to trace the one that works best in predicting postpartum hemorrhage (estimated blood loss greater than 1000 mL) at labor admission. Fifty-five probable risk factors were evaluated, and the model performance was assessed by C-statistics, calibration and decision curves; 152279 births were taken into account, of which 7279 faced postpartum hemorrhage. Results indicated that the Gradient Boosting model performed best (C statistic: 0.93; 95% CI: 0.92–0.93), while the Random Forest model also achieved satisfactory results (C statistic: 0.92; 95% CI: 0.91–0.92). Meanwhile, the two basic statistical models performed slightly worst but overall showed good predicting ability (for the lasso regression model, C statistic: 0.87; 95% CI: 0.86–0.88; and for the logistic regression, C statistic: 0.87; 95% CI: 0.86–0.87). It is clear that ML predictive models can later on be implemented and assist obstetricians in accurately predicting PPH.

### Neonatal mortality prediction

Alongside maternal related issues, Slattery et al.^20^ developed an ML-based classification model using the children’s hospital neonatal database in order to predict neonatal mortality related to hypoxic-ischemic encephalopathy (HIE); 52 non-anomalous neonates with HIE (receiving therapeutic hypothermia as a treatment) were the participants of this study of which 36 survived (69%) and 23 were facing severe HIE (44%). Neural networks (convolutional and two recurrent ones) were employed in an effort to forecast mortality. Results showed that the median specificity of convolutional networks was 81%, whereas lower percentages were obtained concerning recurrent models with long short-term memory (69%) and gated recurrent model units (65%), suggesting that convolutional networks can later on become extremely useful inpatient evaluation and risk classification.

Rittenhouse et al.^21^ created another ML-based model, aiming to identify preterm newborns in underprivileged areas, where there is a high rate of relevant neonatal mortality. Researchers employed ML algorithms as well as maternal factors related to SGA (Small for Gestational Age). Results revealed a set of characteristics (six in particular), both maternal and newborn related, which could eventually lead to precise identification and improved and timely clinical intervention, reducing the problem in LMICs (low- and middle-income countries).

### Fetal monitoring

The use of ML techniques has also made a good impression in fetal cardiology monitoring, a quite demanding task, considering the small size of the fetus heart, its constant moving or even the lack of compatibility that several sonographers present with fetal echocardiography^22^. Nowadays, for instance, ML is widely used for the diagnosis of fetal hypoxia or acidemia based on cardiotocography (CTG) alongside other issues, like prediction of preterm births^21,23^, the risk stratification of chromosomal aneuploidies^24^ or even the prediction of multiple perinatal outcomes^25^. Regarding the connection of ML and CTG, however, it is clear that further research is needed in order to exhaust all ML features and reduce the high inter- and intra-observer variability and decreased accuracy of CTG assessment^22^. To date, many researchers have tried to tackle this issue by employing ML and DL techniques with the help of ANN, SVM and RF, starting from Bassil el al.^26^. Even though there is still plenty of work to be done, Yeo et al.^27^ proposed the FINE method to trace abnormal fetal cardiac anatomy based on automatic echocardiography views (a method which revealed 4 correctly positive cases of abnormality), while Arnaout et al.^28^ employed a convolutional DL method, using 685 echocardiograms, in order to trace the 5 most substantial images of fetus heart, but also to separate and calculate cardiac structures. As a result, fetus hearts were labeled as normal or with a tetralogy of Fallot (TOF) and hypoplastic left heart syndrome. The outcome of the study indicated that the highest specificity (100%) and sensitivity (90%) occurred in the distinction of hypoplastic left heart syndrome and normality. In general, therefore, it is clear that the results of the aforementioned studies inspire hope for future integration of ML models in clinical practice.

In another attempt to integrate ML techniques in the field, Escobar et al.^29^ released an article describing a predictive model that would be able to trace both obstetrical and fetal complications timely, using automated electronic medical record (EMR) data. Derivation and validation datasets were developed during the clinical testing phase, using data from 303678 admissions and 239526 eligible patients. Two sub-models were created initially, one gradient boosting-based and one logistic. Of the two models, the first was eventually rejected because even though it manifested slightly higher levels of accuracy, it presented a non-compatibility with the present-day version of Epic EMR. Therefore, the American integrated managed care consortium Kaiser Permanente Northern California (KPNC), chose to test the second model, which however exhibited inferior results compared to previously developed models for medical use^30-32^. These results pose a hurdle for further clinical trials, as the models’ goal is to serve patients’ best interest. It should be noted, however, that none of the previously developed early warning systems resembles at a high degree the present model (being automatic, returning discrete probability estimates), making the comparison uneven and the existence of new, synergetic structures essential.

In another study, Arnaout et al.^28^ developed an ML model that aimed to identify the five canonical screening views of the fetal heart and to segment cardiac structures to calculate fetal cardiac biometrics. They trained their model in order to distinguish normal hearts, tetralogy of Fallot (TOF) and hypoplastic left heart syndrome (HLHS). The model was then evaluated, returning an overall sensitivity of 75% and a specificity of 76% when distinguishing normal heart versus TOF, while distinguishing normal versus HLHS achieved a sensitivity of 100% and specificity of 90%, both well above average diagnostic rates for these lesions.

### Preeclampsia

Jhee et al.^33^ focused on a single pregnancy complication, namely late-onset preeclampsia, a condition with high percentages of maternal and fetal morbidity and mortality. Due to the fact that there is no way to prevent its occurrence, prediction is necessary for accurate patient monitoring and counseling. The investigators developed a whole series of models, exploiting ML techniques in order to find the one that best operates in such cases; 11006 expecting women who were treated at Yonsei University Hospital participated in the study. Data retrieved from EMRs (from early second trimester to 34 weeks) were employed, in order to forecast preeclampsia occurrence after the 34th week of pregnancy. Meanwhile, pattern recognition and cluster analysis revealed the crucial variables that formed the prediction models, which were in turn constructed on the basis of logistic regression, DT, Naïve Bayes classification, SVM, RF algorithm, and stochastic gradient boosting (SGB) methods. Even though the study did not include, for the most part, first trimester data, as well as multiple incidents of preeclampsia, the SGB model proved to be adequate and showed the best performance compared to others, with an accuracy of 0.973 and false-positive rate of 0.009, rendering it a promising tool for future practical use.

AI and ML techniques monopolized also the 2018 annual congresses of the American Society for Reproductive Biology and European Society for Human Reproduction and Embryology, where researchers shared some interesting models which could eventually be employed in the field of reproductive medicine. In particular, Kort et al.^34^ developed a predicting model regarding blastocyst formation using oocyte mechanical properties. It was discovered that when age, k1 and eta1 algorithms and architecture were employed, the model could accurately forecast blastocyst development with a positive predictive value of 80% (95% CI: 60.45–91.28) and a negative predictive value of 63.8% (95% CI: 53.42–73.18). Meanwhile, Iwata et al.^35^ proposed a DL-based prediction model concerning the identification of good quality embryos. Investigators used images of human embryos acquired from high-resolution time lapse cinematography (31 hourly images recorded for 30 hours). By using the Keras neural network library framework, they managed to get 94% accuracy for the training dataset and 70% for the validation dataset. However, after 50 learning sessions, the relevant values were 92% and 80%, respectively. Finally, Tran et al.^36^ employed AI techniques in an effort to select proper embryos. Using the AI deep neural network, they achieved a Mean Area Under the Curve (AUC) of 0.93 (95% CI: 0.92–0.94) regarding the prediction of fetal heartbeat outcome on the validation dataset.

### Dystocia

Bartal et al.^37^ proposed an ML model in order to forecast shoulder dystocia (SD) or other birth injuries in women proceeding with vaginal delivery, by using maternal demographics, obstetric history, and sonographic evaluation (5 weeks from delivery). Between January 2013 and June 2019, a total of 490 patients were included in the study [non-anomalous singleton pregnancies with a sonographic estimated fetal weight (EFW) larger or equal to a 35-week pregnancy]. In the sample group, 381 women (77.8%) proceeded with vaginal delivery and 109 (22.2%) had a cesarian delivery because they were diagnosed with suspected fetal macrosomia. In the meantime, SD or other birth injuries occurred 19 times. Overall, results indicated that EFW assessment alone produced inferior outcomes compared to the combination of ML and EFW (AUC: 0.61 vs 0.77), while in the second case, the assessment of multiple risk scores was rated >0.5, suggesting that further research on the area could eventually prove beneficial in preventing SDs and other birth injuries, as well as reducing related neonatal morbidity and the choice of cesarian delivery due to fear of SD occurrence during VD.

### Predicting successful vaginal deliveries

In a similar study, Guedalia et al.^38^ developed another ML model in order to predict successful vaginal deliveries. Achieving a successful vaginal delivery is a matter of crucial importance, taking into consideration that the health and the development of the child are highly influenced by the quality of delivery^38^. However, existing monitoring methods are facing difficulties distinguishing the cases where vaginal delivery would be dangerous and cesarian delivery would be necessary, leading to an increased number of cesarian deliveries throughout the world. By using a personalized ML-based prediction model and real-time data of the first stage of labor, researchers tried to address this issue. Overall, 94480 cases of vaginal delivery were studied. Three sub-models were created, namely one that used data extracted only at the time of admission, one that used the first examination’s real-time cervical data, and one that also included data from the end of the first stage of labor. The third sub-model proved to be more efficient with an AUC of 0.917 (95% CI: 0.913–0.921), while the previous two (with data only from the time of admission and data extracted from the first cervical examination) achieved an AUC of 0.817 (95% CI: 0.811–0.823) and 0.819 (95% CI: 0.813–0.825), respectively. Therefore, it is clear that further research and upgrading of personalized ML-based prediction models is necessary, as these models could eventually assist medical practitioners in decision-making with regard to means of delivery, and avoiding a great number of non-required CDs.

Lipschuetz et al.^39^ proposed a model in order to predict the chance of a successful vaginal delivery (VD) after the occurrence of a cesarian delivery (CD). These cases are commonly encountered, as many women decide to attempt a vaginal delivery in order to soothe the load coming from a previous CD. A developed model, however, should achieve great accuracy in predicting successful VDs, as an unplanned cesarian delivery of the last-minute exhibits more complications; 9888 women participated in the specific study, meeting the criteria regarding the existence of a previous CD, while 75.6% of the sample attempted a natural delivery with a relatively high success rate of 88%. Two ML based sub-models were created (one with data gathered from the first antenatal visit and another with added data available close to the delivery process). The second model exhibited better results than the first (AUC=0.793; 95% CI: 0.778–0.808 vs AUC=0.745; 95% CI: 0.728–0.762), suggesting that it could eventually be integrated in clinical practice, preventing the repeated occurrence of CDs.

## CONCLUSIONS

ML algorithms have given hope regarding the solution of many unresolved issues in the field of midwifery and obstetrics. Nevertheless, there is still much work to be done, in order to be able to fully exploit the potential of ML and decrease the flaws that may occur, while moving from theory to practice^2^. Such flaws could affect the trust of both experts and patients to ML practices, while carrying legal and ethical implications too. For this reason, it is evident that experts should be held responsible for the outcomes of the models they design and evaluate, always keeping in mind that in order for these models to operate accurately, large, high-quality training datasets are required, as small or poor-quality ones could lead to inferior outcomes^2^. Despite the multiple issues that need to be resolved though, it is evident that ML has the potential to help in a variety of clinical domains in obstetrics, and therefore researchers should continue to explore their vast potential.

## Data Availability

Data sharing is not applicable to this article as no new data were created.
